# Dlaczego Szczepienia Ochronne są Nadal Potrzebne?

**DOI:** 10.34763/devperiodmed.20182202.171178

**Published:** 2018-06-30

**Authors:** Hanna Czajka

**Affiliations:** 1 Wydział Medyczny Uniwersytetu Rzeszowskiego w Rzeszowie, Polska; 2 Wojewódzki Specjalistyczny Szpital Dziecięcy im św. Ludwika w Krakowie, Polska

**Keywords:** szczepienia, choroby zakaźne, ruchy antyszczepionkowe, rodzice, odmowa, edukacja, vaccinations, infectious diseases, anti-vaccine movements, parents, refusal, education

## Abstract

Szczepienia ochronne w Polsce i innych krajach uprzemysłowionych stają od przeszło dwóch dekad w ogniu krytyki ze strony środowisk jawnie niechętnych tej formie profilaktyki chorób zakaźnych. Wygłaszane przez aktywistów ruchów antyszczepionkowych oraz wyznawców medycyny naturalnej argumenty oraz zarzuty przeciw szczepieniom dzieci i młodzieży oraz szczepionkom, jako lekom, nie są oparte na wiarygodnych danych medycznych. Wbrew kłamliwym teoriom zagrożenie ze strony chorób zakaźnych nie jest wcale mniejsze niż w przeszłości, co więcej wzrasta ono w ostatnich latach wskutek zwiększania się liczby dzieci uchylających się od realizacji szczepień. Pomimo to, błędne argumenty trafiają na podatny grunt podważając zaufanie do szczepień i szczepionek wśród rodziców lub opiekunów dzieci objętych programem szczepień. W tej sytuacji obowiązkiem lekarzy sprawujących opiekę profilaktyczną nad dziećmi, a także odpowiedzialnych za realizację szczepień organów administracji, uczelni medycznych i innych instytucji ochrony zdrowia, jest podniesienie poziomu edukacji społecznej na temat szczepień, a z drugiej strony skuteczne przeciwstawianie się propagandzie antyszczepionkowej. Środkiem do osiągnięcia tych celów jest dokładne rozeznanie potrzeb społecznych oraz stałe podnoszenie kwalifikacji fachowych lekarzy POZ, położnych, pielęgniarek środowiskowych oraz lekarzy innych specjalności, którzy mają codzienny kontakt z niemowlętami i ich rodzicami. Temu celowi powinno służyć także położenie większego nacisku na problem profilaktyki chorób zakaźnych na wszystkich kierunkach uczelni medycznych.

## Abstract (en)

Vaccinations in Poland and other industrialized countries have been in the firing line for more than two decades. Members of the ”anti-vaccine movement” and alternative medicine supporters are openly reluctant to this form of preventing infectious diseases, however their arguments are not based on reliable medical data. Contrary to false theories, the threat of infectious diseases is not lower than in the past. What is more, it has escalated in recent years as a result of the increasing number of parents who avoid vaccinations. Nevertheless, erroneous arguments find a breeding ground among parents or guardians of children covered by vaccination programs undermining trust in vaccines in general. In this situation, it is the responsibility of: physicians who provide preventive care for children, administrative bodies responsible for implementing immunization but also medical schools and other healthcare institutions, on the one hand to raise the level of social awareness about the meaning of vaccination, and on the other to effectively oppose the anti-vaccine propaganda. The way to achieve these goals is to thoroughly understand social needs and constantly improve the professional qualifications of primary care physicians, midwives, community nurses and doctors of different specialties who have daily contact with children and their parents. The key area of focus should also be to place greater emphasis on education about the prevention of infectious diseases among students of all medical school faculties.

## Wstęp

Szczepienia ochronne są procedurami medycznymi mającymi znaczący wpływ na zdrowie dzieci oraz ochronę przed chorobami zakaźnymi. Stanowią jedno z podstawowych ogniw wielokierunkowej polityki zdrowotnej państwa. Nasz Program Szczepień Ochronnych (PSO) obejmuje szczepienia obowiązkowe dzieci, młodzieży oraz osób szczególnie narażonych na zakażenia. Ta część szczepień wykonywana jest w systemie scentralizowanym. Natomiast szczepienia zalecane, nie finansowane z budżetu Państwa, są realizowane w systemie zdecentralizowanym. Obowiązek poinformowania o szczepieniach z obu tych grup spoczywa na barkach lekarza podstawowej opieki zdrowotnej. Sprawne przeprowadzanie szczepień masowych przyczynia się do wytworzenia odporności populacyjnej, a tym samym do ograniczenia liczby zachorowań na daną chorobę. Wprowadzenie powszechnych szczepień obowiązkowych i konsekwentne egzekwowanie tego obowiązku przez nadzór sanitarno-epidemiologiczny pozwoliło ograniczyć zapadalność na wiele chorób zakaźnych, w tym na błonicę, tężec, wirusowe zapalenie wątroby typu B czy poliomyelitis. Wspólnym sukcesem krajowych programów szczepień, koordynowanych przez World Health Organization (WHO) była światowa eradykacja ospy prawdziwej w roku 1979. Ważnym czynnikiem wpływającym na profilaktykę chorób zakaźnych jest postrzeganie szczepień przez społeczeństwo. Znaczącą rolę odgrywają w tej dziedzinie media, choć sposób przedstawiania problematyki szczepień w prasie, stacjach radiowych czy telewizyjnych nie zawsze jest właściwy i zgodny z aktualną wiedzą medyczną. Prezentowane informacje często kojarzą szczepienia z wieloma chorobami będącymi ubocznymi skutkami immunizacji. Podnoszenie poziomu wiedzy i świadomości społecznej na temat szczepień poprzez środki masowego przekazu oraz intensywniejsze prowadzenie szkoleń z tej dziedziny dla obecnych i przyszłych pracowników ochrony zdrowia powinno przeciwdziałać obniżaniu się liczby osób odmawiających realizacji szczepień ochronnych.

## Dlaczego szczepienia są nadal potrzebne?

Aby odpowiedzieć na to pytanie, należy przyjrzeć się danym epidemiologicznym kilku wybranych chorób, którym zapobiegamy poprzez szczepienia.

### Gruźlica

*Mycobacterium tuberculosis* – bakteria powodująca zachorowania na gruźlicę przenosi się głównie drogą kropelkową od osoby z aktywną postacią choroby. Na uwagę zasługuje fakt, że tylko 5-10% osób które zostały zakażone rozwija aktywną postać choroby w ciągu dwóch lat od ekspozycji. Gruźlica stanowi jednak globalny problem zdrowotny i corocznie na świecie odnotowuje się 9 milionów nowych zachorowań i 1,8 mln zgonów z jej powodu [1].

Najczęstszą postacią choroby jest gruźlica płuc. Specyficzną odmianą choroby jest gruźlica wielolekooporna (MDR). Bakteria może zaatakować praktycznie każdy narząd lub wiele z nich jednocześnie (postać rozsiana). Szczepienie BCG (*Bacillus Calmette-Guérin*) zapobiega zachorowaniom na ciężkie i potencjalnie śmiertelne postaci gruźlicy u małych dzieci. Szczepienie powinno odbyć się jak najwcześniej po urodzeniu – najpóźniej przed wypisem dziecka z oddziału noworodkowego. Szczepienie BCG jest skuteczne w zapobieganiu gruźliczemu zapaleniu opon mózgowo-rdzeniowych oraz gruźlicy prosówkowej u małych dzieci.

Zapadalność na gruźlicę w Polsce wynosi 16,7 przypadków na 100 tys. ludności. Zgodnie z definicją *European Centre for Disease Prevention and Control* (ECDC) zapadalność niższą niż 20 zachorowań na 100 tys. ludności określa się jako niską, a dopiero przy spadku zapadalności poniżej 10 osób na 100 tys. mówimy o zbliżaniu się do fazy eliminacji choroby. Taka sytuacja występuje w 22 krajach Europy. W Polsce na gruźlicę chorują przede wszystkim osoby starsze, które z chorobą zetknęły się w młodości, a zachorowania mają związek ze spadkiem odporności w podeszłym wieku. Zachorowania u dzieci i młodzieży po wprowadzeniu szczepień obowiązkowych występują rzadko. Po raz ostatni w Polsce trzy zgony z powodu gruźlicy u dzieci poniżej 14. roku życia odnotowano w 2011 roku, a przed wprowadzeniem w naszym kraju szczepień powszechnych przeciw gruźlicy w połowie lat 50. XX wieku śmiertelność była bardzo wysoka, dla przykładu w 1950 r. z powodu gruźlicy zmarło ponad 3 tys. dzieci w wieku poniżej 14. roku życia [2].

Nie należy jednak zapominać, że za naszą wschodnią granicą sytuacja epidemiologiczna gruźlicy kształtuje się znacznie mniej korzystnie. Według danych *World Health Organization* (WHO) przytaczanych w raporcie ECDC z roku 2017 liczba zachorowań na gruźlicę w Rosji wynosi rocznie 115 tysięcy, na Ukrainie 41 tysięcy, a w należącej do Unii Europejskiej Rumunii 16 000. Najwyższa zapadalność na tę chorobę jest odnotowywana w Mołdawii 152/100 000, Gruzji 99/100 000 i na Ukrainie 91/100 000 mieszkańców. Zapadalność na gruźlicę na Białorusi, wg danych ECDC za rok 2015, wynosiła 44/100 000, a wskaźniki śmiertelności z powodu gruźlicy w Rosji i na Ukrainie wynoszą po 11/100 000 mieszkańców [3].

Wirusowe zapalenie wątroby typu B (WZW B - HBV)

Zakażenie wirusem zapalenia wątroby typu B (HBV) może prowadzić do stałego nosicielstwa tego wirusa i być przyczyną przewlekłego zapalenia wątroby, marskości wątroby i pierwotnego raka wątroby. Wysokie wskaźniki nosicielstwa u kobiet związane są z częstszym zakażeniem WZW typu B niemowląt i młodszych dzieci. Najczęściej do zakażenia dochodzi przez przerwanie ciągłości tkanek i wprowadzenie wirusa do organizmu poprzez niesterylne narzędzia. Zakażenie jest możliwe także podczas zabiegów kosmetycznych oraz w wyniku kontaktów seksualnych z zakażoną osobą.

Ocenia się, że głównym źródłem zachorowań na WZW B były zakażenia w placówkach ochrony zdrowia, a odsetek zachorowań wśród małych dzieci wynosił 80%. Rejestracja zachorowań na WZW B jest prowadzona od 1979 roku. W latach 1977-1985 odnotowywano rocznie przeciętnie 15 785 zachorowań, przy zapadalności 43,6/100 000. W kolejnych latach odnotowano niewielki spadek zachorowań do 14,7 tysięcy rocznie, przy zapadalności 40,3/100 000. Zapadalność na WZW B w Polsce była wówczas najwyższa w Europie. Po 1985 roku nastąpiła poprawa tych wskaźników, wiązana z poprawą sterylizacji sprzętu medycznego. Znaczący spadek odnotowywany od 1993 roku wynikał z wdrożenia programu szczepień przeciw WZW B w grupach ryzyka, a od 1996 r. szczepienia obowiązkowe objęły wszystkie noworodki i niemowlęta w naszym kraju. W tym samym roku zapadalność wynosiła 16,7/100 000, a w roku 1999 już tylko 9,1/100 000 [2]. Zbierane przez Narodowy Instytut Zdrowia – Państwowy Zakład Higieny w Warszawie dane epidemiologiczne, dotyczące liczby zachorowań na ostre wirusowe zapalenie wątroby typu B, utrzymują się na przestrzeni ostatnich lat na zbliżonym poziomie. W roku 2017 odnotowano w naszym kraju 55 zachorowań na ostre WZW B przy zapadalności 0,14/100.000 osób. Rok wcześniej liczba zachorowań wyniosła 50, a zapadalność 0,13/100 000. Należy także zauważyć, że w grupie kobiet w wieku rozrodczym ostre WZW B w roku 2016 rozpoznano u 6 kobiet w wieku 30-44 lat, natomiast w grupie Polek w wieku poniżej 30 lat nie odnotowano żadnego przypadku tej choroby [4]. Stan taki, jak już wspomniano, zawdzięczamy wprowadzonym w 1996 r. obowiązkowym szczepieniom wszystkich noworodków przeciw zakażeniom HBV, a od 2000 roku szczepieniami wyrównawczymi przeciw WZW B objęto także młodzież w wieku gimnazjalnym.

### Krztusiec

Krztusiec – zakaźna choroba bakteryjna wywoływana przez *Bordetella pertussis* występuje w całym świecie. W przebiegu klinicznym choroby wyróżniamy trzy fazy – nieżytową, napadowego kaszlu i zdrowienia. Ciężkie postaci krztuśca występują u najmłodszych niemowląt w 1. półroczu życia, w tej grupie chorujących przebieg może być nietypowy, z bradykardią, bezdechem, ale bez typowego, napadowego kaszlu. W grupie niemowląt występują częściej powikłania w postaci zapaleń płuc, drgawek, encefalopatii, bezdechów i zgonów. Ponad 2/3 niemowląt z krztuścem wymaga hospitalizacji.

Wprowadzenie w połowie XX wieku szczepień przeciw krztuścowi (w Polsce od roku 1960) spowodowało redukcję zachorowań i do roku 1990 dane epidemiologiczne świadczyły o wysokiej skuteczności szczepionki. Od 1990 roku obserwuje się jednak wzrost zapadalności na krztusiec, w tym wśród szczepionych dzieci w wieku powyżej 5. roku życia, wśród nastolatków i dorosłych. Stanowią oni źródło zakażenia dla nieuodpornionych niemowląt, dla których choroba jest szczególnym zagrożeniem [2].

Ostatni znaczący wzrost zachorowań w Polsce wystąpił w roku 2016, kiedy zanotowano 6856 zachorowań [5]. Prawdopodobnymi przyczynami wzrostu liczby zachorowań na krztusiec są zanikanie odporności poszczepiennej, jak również odporności po przechorowaniu krztuśca, podatność niemowląt na zachorowania na krztusiec, lepsze metody diagnostyki krztuśca, sprawniejszy nadzór epidemiologiczny, krążenie zmodyfikowanych szczepów *Bordetella pertussis*, mniejsza skuteczność szczepionek z acelularnym komponentem krztuśca czy wreszcie spadek odsetka szczepionych dzieci spowodowany wzrostem liczby odmów ze strony rodziców [6].

### Odra

Odra jest ostrą chorobą wirusową. Zakaźność odry jest bardzo duża. Chory jest zakaźny dla otoczenia na 3-5 dni przed wysypką i przez pierwsze 3 dni wysypki. Do zakażenia dochodzi drogą kropelkową, a źródłem zakażenia jest człowiek. Okres wylęgania wynosi 9-11 dni, a w przebiegu klinicznym odry wyróżnia się okres nieżytowy, wysypkowy i okres zdrowienia. Okres nieżytowy trwa 3–4 dni i obejmuje: wysoką gorączkę, kaszel suchy, męczący, zapalenie gardła, katar, zapalenie spojówek ze światłowstrętem. Po 3-4 dniach okresu nieżytowego pojawiają się na błonie śluzowej policzków białe plamki otoczone czerwoną obwódką (plamki Koplika), które znikają po wystąpieniu wysypki. Powikłaniami odry są zapalenie ucha środkowego (7-9%), zapalenie płuc (1-6%), biegunka (8%), zapalenie mózgu (1/1000 – 2000 przypadków), albo podostre stwardniające zapalenie mózgu (SSPE 1/100000). Najwięcej zgonów oraz najwyższa umieralność występuje wśród dzieci w wieku do 12. miesiąca życia [7].

Prowadzone od lat szczepienia przeciw wirusowi odry spowodowały znaczący spadek liczby zachorowań w krajach realizujących profilaktykę czynną. W 1975 roku w Polsce szczepienie przeciw odrze wprowadzono do Programu Szczepień Ochronnych dla dzieci w wieku 13-15 miesięcy. Po wprowadzeniu profilaktyki odsetek zaszczepionych dzieci stale wzrastał od 50% podlegających obowiązkowi szczepień do 79% w roku 1988. Od wprowadzenia szczepień obowiązkowych zapalność na odrę stopniowo malała, choć w roku 1984 oraz w roku 1990 wystąpiły w Polsce dwie duże epidemie odry z zapadalnością 148/100 000. Zachorowania odnotowywano przede wszystkim w grupie nastolatków, stąd dla uniknięcia kolejnych epidemii wprowadzono drugą dawkę szczepień przeciw odrze w 7. roku życia [2]. W danych epidemiologicznych odry publikowanych w opracowaniach Państwowego Zakładu Higieny Narodowego Instytutu Zdrowia w Warszawie wynika, że liczba zachorowań na odrę ulega okresowych wahaniom. Dla przykładu w roku 2016 odnotowano w Polsce 133 przypadki odry (zapadalność 0,35/100 000), podczas gdy rok wcześniej w 2015 roku zachorowań na odrę było w Polsce tylko 48 (zapadalność 0,12/100 000).

Choć sytuacja epidemiologiczna w Polsce jest dość stabilna, to jednak w niektórych państwach Europy odnotowujemy epidemie tej choroby. Od lutego 2016 występują masowe zachorowania na odrę w Rumunii. Pomiędzy 01.01.2016 a 30.06.2017 odnotowano w Rumunii 7491 przypadków odry – w tym 31 zgonów z jej powodu. We Włoszech w 2017 roku zachorowało na odrę w okresie od stycznia do lipca 3346 osób, z czego dwie zmarły. Wśród osób chorujących było 252 pracowników ochrony zdrowia, a średni wiek chorujących wynosił 27 lat. Spośród wszystkich zachorowań 88% przypadków dotyczyło osób wcześniej nie szczepionych przeciw tej chorobie.

We Francji w pierwszym półroczu 2017 odnotowano 295 zachorowań na odrę, co stanowiło 6-krotny wzrost w stosunku do zachorowań z pierwszego półrocza 2016 roku. Najczęściej chorowały dzieci w 1. roku życia, odnotowano 2 przypadki odrowego zapalenia mózgu oraz 22 przypadki ciężkiego odrowego zapalenia płuc.

Najbardziej niepokojący dla nas jest rejestrowany w 2018 roku wybuch epidemii odry na Ukrainie. W ciągu pierwszych dwóch tygodni tego roku zanotowano 1285 przypadków choroby, w tym 856 zachorowań wśród dzieci. W 2017 roku stwierdzono na Ukrainie 4782 zachorowania na odrę, w tym 5 zgonów [8].

## Wpływ prawidłowej realizacji pso na sytuację epidemiologiczną

Wraz ze wzrostem liczby osób uodpornionych w populacji zmniejsza się szansa na zachorowanie na daną chorobę przez osobę nieuodpornioną. W zależności od specyfiki drobnoustroju odporność gromadna najczęściej pojawia się po zaszczepieniu ponad 80% populacji. O odporności zbiorowiskowej możemy mówić w przypadku tych chorób zakaźnych, których czynnik etiologiczny jest szeroko obecny w środowisku i przenosi się z człowieka na człowieka. Próg odporności zbiorowiskowej dla takich chorób jak odra czy krztusiec wynosi 92-95% osób uodpornionych w populacji i oznacza, że po osiągnięciu takiego odsetka szczepionych liczba nowo zakażonych osób zaczyna się zmniejszać [9].

W niektórych krajach świata obserwuje się obniżenie odsetka osób uodpornionych. Dane pochodzące ze Światowej Organizacji Zdrowia (WHO) za rok 2016 pokazują spadek pokrycia szczepionkowego drugą dawką szczepionki przeciw odrze poniżej 95% w 20 z 27 krajów europejskich, a pokrycie pierwszą dawką tej szczepionki poniżej 95% w 18 z 30 krajów Europy [10]. W Polsce stan zaszczepienia dzieci przeciw odrze, śwince, różyczce (MMR) jest wysoki. Dla dzieci w 3. roku życia wynosił w roku 2015 − 96,3% a w roku 2016 − 95,5%. Nieco gorszy jest odsetek zaszczepionych w Polsce dzieci w 11. roku życia drugą dawką szczepionki MMR, który w 2015 r. wyniósł 94,1%, a w roku 2016 odpowiednio 93,4%. Choć nie są to jeszcze dane alarmujące, to jednak obserwuje się niewielki spadek odsetka wyszczepialności w poszczególnych rocznikach, co przy wzrastającej liczbie rodziców odmawiających szczepienia dzieci może budzić niepokój. W roku 2007 odmówiło wykonania szczepień u dzieci 4893 rodziców lub opiekunów, a w roku 2016 liczba ta wzrosła do 23 147 osób [11].

## Ruchy antyszczepionkowe

Od przeszło dwóch dekad rośnie w krajach uprzemysłowionych liczba rodziców, którzy odmawiają zgody na wykonanie szczepień ochronnych u swych dzieci. Sceptyczne i krytyczne opinie towarzyszą szczepieniom od chwili ich wprowadzenia. Po raz pierwszy pojawiły się one w Anglii w roku 1853 w reakcji na pierwsze szczepienia przeciw ospie prawdziwej podejmowane przez Jennera [12]. Krytykowane przez niektóre gremia działania Jennera zapoczątkowały rozwój wakcynologii, doprowadziły do eradykacji ospy prawdziwej na świecie, potwierdzonej przez WHO w dniu 09 grudnia 1979 roku.

Pod koniec XIX w. ruchy antyszczepionkowe pojawiły się w Stanach Zjednoczonych, co doprowadziło do zniesienia obowiązku szczepień w niektórych stanach, a w konsekwencji tej decyzji w latach 1901-1903 w Bostonie i okolicach wystąpiła epidemia ospy prawdziwej. Po wprowadzeniu w drugiej połowie XX wieku szczepień przeciw krztuścowi szybko uzyskano ograniczenie liczby przypadków krztuśca, ale w latach 70. XX. wieku w Wielkiej Brytanii pojawiły się doniesienia o encefalopatii związanej ze szczepieniami przeciw krztuścowi. Ten zarzut sformułowany przez Stewarda został obalony na podstawie wyników dużego badania epidemiologicznego, co pozwoliło na ponowne rozpoczęcie kampanii propagowania tych szczepień. W latach 80. XX w. w Stanach Zjednoczonych pojawiły się kontrowersyjne doniesienia na temat szczepionki DTP. W 1998 roku wybuchła kolejna afera sugerująca związek szczepionki MMR z autyzmem dziecięcym, wysunięta przez doktora Andrew Wakefielda. Opublikowany w czasopismie *Lancet* artykuł jego autorstwa odbił się szerokim echem w świecie, a w efekcie odnotowano znaczący spadek liczby szczepień w Stanach Zjednoczonych i Wielkiej Brytanii, skutkujący następnie znaczącym wzrostem liczby zachorowań na odrę i świnkę. Przeprowadzone badania epidemiologiczne, biologiczne i kliniczne wykazały brak związku przyczynowego pomiędzy szczepieniem MMR a występowaniem autyzmu. W roku 2010 wycofano publikację Weakfielda z czasopisma *Lancet*, a jej autorowi, na podstawie zarzutu nieetycznego postępowania i fałszowania wyników badań, odebrano prawo wykonywania zawodu lekarza.

Kolejny zarzut postawiono tiomersalowi, stosowanemu w szczepionkach jako środek konserwujący. Także i w tym wypadku badania kliniczne i epidemiologiczne nie potwierdziły prawdziwości tych doniesień. Pomimo odparcia ww. zarzutów wobec szczepień i szczepionek, oskarżenia te nie zniknęły z przestrzeni publicznej. Trafiły na podatny grunt, są nadal wykorzystywane z pogwałceniem zasad EBM przez przeciwników szczepień, próbujących nadal niesłusznie podważać rzetelność i uczciwość badań naukowych oraz programów szczepień [13].

Oceniając ten problem z perspektywy historycznej, trzeba zwrócić uwagę na regułę, zgodnie z którą dopuszczenie do powszechnego stosowania szczepionki przeciw chorobie, wobec której byliśmy dotąd bezradni, powoduje na pierwszym etapie wyraźny efekt pozytywany – spadek liczby zachorowań. Następnie po pewnym czasie zaczyna się akcentować niepożądane efekty stosowania tego leku. Przy spadku zagrożenia chorobą skutkuje to obniżeniem się poziomu realizacji szczepień i po jakimś czasie choroba powraca [14].

Główne teoretyczne założenia ideologii antyszczepionkowej w XIX i XX wieku sprowadzały się do następujących haseł:

- szczepionki powodują chorobe iteopatyczną;
- propogowanie szczepien to bezbozny sojusz producentów i lekarzy, motywowany żądzą zysku ze sprzedaży szczepionek;
- szczepionki to trujące koktajle chemiczne;
- zwolennicy szczepien boją sie „poszukiwania prawdy″, dlatego ukrywają błędy i zamykają swe oczy na fakty, a także nie odpowiadają na zadawane im pytania;
- prawny obowiązek realizacji szczepien stanowi obelgedla każdego podmiotu sfery publicznej,
- odpornosc na szczepionke jest tymczasowa;
- alternatiwą dla szczepien jest zdrowy tryb zycia, higiena osobista i przestrzeganie diety, co zabezpiecza przed zachorowaniem [12].

Cenne informacje na temat przeciwników szczepień można znaleźć w opublikowanym przez Biuro Regionalne dla Europy Centralnej i Wschodniej UNICEF w kwietniu 2013 roku opracowaniu pt.: „Sledzenie nastrojow antyszczepionkowychwe wschodnioeuropejskich sieciach społecznościowych” (*Tracking Antivaccination Sentiment in Estern European Social Media Network*) [15]. W opracowaniu znajdują się, między innymi, dane dotyczące naszego kraju.

Spośród polskich antyszczepionkowców 59% to kobiety, a mężczyźni stanowią 41%. Najwięcej przeciwników szczepień rekrutuje się z grupie wiekowej 30-40 lat (32%), w pozostałych czterech grupach wiekowych (10-20; 20-30; 50-60 i 70-80) rozkład przeciwników szczepień jest równy po 17% w każdej z nich. Przeciwnicy szczepień w Polsce, wykorzystując Internet do propagowania swych idei, prezentują swe poglądy na specjalistycznych blogach (85%) oraz za pośrednictwem Facebooka (13%). Pozostałe 2% poglądów antyszczepionkowych ujawniana jest w tzw. newsach, na portalu Twitter oraz na innych forach internetowych.

Przy propagowaniu sztandarowych haseł ruchów antyszczepionkowych podział ich zwolenników ze względu na płeć wskazuje, że kobiety preferują poglądy bardziej racjonalne. Stanowią bowiem wyraźną mniejszość wśród zwolenników teorii spiskowej (37%) czy w grupie motywowanej względami religijnymi lub etycznymi (42%), a także wśród przeciwników szczepień motywowanych brakiem zaufania do szczepionek (48%). Natomiast kobiety stanowią większość w gronie przeciwników szczepień wskazujących jako przyczynę swych poglądów zagrożenie efektami ubocznymi szczepień (54%), określających szczepionki jako szkodliwe związki toksyczne lub chemiczne (56%), oraz uzasadniających uchylanie się od szczepień obawą przez zaburzeniami rozwojowymi dziecka (59%). Najbardziej niepokojące jest jednak to, że w gronie przeciwników szczepień można spotkać także lekarzy i innych pracowników ochrony zdrowia.

## Informacja o szczepieniach a rozmowa z lekarzem

Wobec powszechnego w skali światowej wzrostu liczby rodziców/opiekunów dzieci odmawiających poddania swych pociech obowiązkowym lub zalecanym szczepieniom ochronnym, zachodzi pytanie, w jaki sposób pediatrzy lub lekarze rodzinni, sprawujący nad dziećmi podstawową opiekę medyczną, powinni rozmawiać na temat szczepień i chorób zakaźnych z rodzicami swych pacjentów.

Interesujących danych o znaczeniu informacji o czynnej profilaktyce chorób zakaźnych, docierających do rodziców lub opiekunów dzieci dostarczyli w 2017 roku naukowcy z Norwegii i Republiki Południowej Afryki w przeprowadzonym pod egidą *Cochrane Collaboration* przeglądzie 38 badań, zbierających w różnych krajach opinie rodziców i opiekunów dzieci w wieku do 6 lat na temat jakości przekazywanych im informacji o rutynowych szczepieniach dziecięcych [16]. *Cochrane Collaboration* jest cieszącą się autorytetem niezależną międzynarodową organizacją typu *non-profit*, której celem jest ułatwianie podejmowania świadomych i trafnych decyzji dotyczących postępowania medycznego poprzez opracowywanie dowodów (opartych na faktach, zgodnych z regułami EBM), które następnie są publikowane w bazach Biblioteki Cochrane (*Cochrane Library*) w formie przeglądów systematycznych.

Autorzy przeglądu potwierdzają, że stosowanie szczepionek pediatrycznych jest skutecznym sposobem zapobiegania poważnym chorobom wieku dziecięcego, ale nie wszystkie dzieci otrzymują zalecane im szczepionki. Jest to spowodowane różnymi przyczynami, wśród nich np. brakiem dostępu do profilaktycznych zabiegów medycznych z uwagi na niewłaściwą organizację opieki medycznej lub zbyt dużymi odległościami pomiędzy miejscem zamieszkania a siedzibami placówek świadczeń zdrowotnych. Zauważalnym zjawiskiem jest także problem finansowy, czyli brak środków na indywidulany zakup szczepionek zalecanych (nie zawsze finansowanych przez państwo lub refundowanych przez system ubezpieczeń zdrowotnych). Inni rodzice mogą nie mieć zaufania do szczepionek jako leków lub nie ufać pracownikom opieki zdrowotnej prowadzącym profilaktykę. Kolejna grupa rodziców lub opiekunów może nie widzieć potrzeby uodparniania swych dzieci z powodu niewiedzy lub błędnych informacji o szczepieniach oraz chorobach którym możemy zapobiegać.

Autorzy opracowania skoncentrowali się w swym przeglądzie na ocenie problemu przekazu informacji na temat szczepień, które docierają do rodziców/opiekunów dzieci w wielu formach i z różnorodnych źródeł. Z naszego punktu widzenia właściwy poziom tej komunikacji jako drogi do zahamowania spadku wyszczepialności dzieci, jest jednym z podstawowych elementów poprawnie realizowanej polityki zdrowotnej. Pracownicy ochrony zdrowia muszą mieć świadomość, że do rodziców ich pacjentów informacje na temat szczepień docierają wieloma drogami, niekoniecznie dającymi możliwość weryfikacji poprzez bezpośrednią wymianę poglądów, stąd najczęściej decyzje odmowne rodziców i opiekunów dzieci są oparte na nieracjonalnych lub pseudonaukowych podstawach.

Rozmowa twarzą w twarz z pracownikiem ochrony zdrowia (lekarzem lub pielęgniarką) jest dla rodziców małych dzieci jedynym kanałem informacyjnym umożliwiającym wymianę poglądów, skonfrontowania korzyści ze szczepień ochronnych z ewentualnymi zagrożeniami związanymi z wakcynacją, czy wreszcie omówienie problematyki chorób zakaźnych, którym zapobiegamy poprzez szczepienia.

Stanowisko autorów przeglądu, kierowane do osób odpowiedzialnych za planowanie działań zmierzających do podniesienia wyszczepialności najmłodszych pacjentów, obejmuje 8 pytań (problemów), dzięki analizie których można ustalić przyczyny negatywnego stosunku do szczepień ochronnych ze strony rodziców/opiekunów dzieci:

1. Czy informacje o szczepieniach są przekazywane rodzicom w odpowiednim czasie przed szczepieniem? Czy rodzice mają wystarczającą ilość czasu na analizę tych informacji i na podjęcie decyzji?
2. Jak szeroko informacje na temat szczepień są dostępne w systemie opieki zdrowotnej? Czy rodzice mają możliwość prowadzenia dyskusji na temat szczepień?
3. Czy informacje na temat szczepień są dostosowane do potrzeb każdej rodziny? Na przykład rodzice niezdecydowani na szczepienie mogą oczekiwać innych informacji niż rodzice akceptujący podanie szczepionek.
4. Czy pracownicy ochrony zdrowia zapewniają lub pomagają odnaleźć rodzicom obiektywne informacje na temat szczepień, dostosowane do ich potrzeb?
5. Czy prowadzą oni z rodzicami w sposób troskliwy, wrażliwy i nieoceniający otwarte, pełne empatii rozmowy? Czy udzielają jasnych odpowiedzi na pytania rodziców? Czy rodzice mają zapewnione przyjazne otoczenie dla podejmowania decyzji?
6. Czy pracownicy służby zdrowia nie są postrzegani jako kierujący się korzyściami finansowymi, zamiast najlepszym interesem dziecka?
7. Czy rodzice oceniają otrzymywane informacje o szczepieniach jako bezstronne, wiarygodne, i zrozumiałe?
8. Czy strategie komunikacji szczepień są wyczulone na konieczność podejmowania zdecydowanej reakcji na doniesienia medialne, pogłoski lub negatywne opinie na temat szczepień, aby w odpowiedni sposób odpowiedzieć na pytania rodziców i rozwiać obawy, które te opowieści mogły wzbudzić?

Dla przykładu w Holandii pierwszą, zindywidualizowaną informację na temat narodowego programu szczepień, rodzice dziecka otrzymują bezpośrednio od położnej odwiedzającej ich w domu, gdy noworodek kończy drugi tydzień życia. Informacja ta jest ponawiana przez właściwe centrum opieki medycznej drogą mailową, gdy dziecko ma 4-6 tygodni. Jego rodzice otrzymują wtedy broszurę z wykazem szczepionek, informacje o chorobach zakaźnych wieku dziecięcego, aktualny program szczepień, informację na temat efektów ubocznych szczepień oraz wskazanie strony internetowej Narodowego Instytutu Zdrowia Publicznego i Środowiska (RIVM). Gdy dziecko kończy 8 tygodni, jego rodzice odwiedzają wraz z nim właściwe centrum opieki pediatrycznej na pierwszej wizycie szczepiennej związanej ze sprawdzeniem stanu zdrowia dziecka. Pomimo to niektórzy holenderscy rodzice twierdzą, że nie uzyskali wystraczających informacji na temat ubocznych efektów szczepień [17].

W naszym systemie opieki zdrowotnej obowiązek poinformowania rodziców dziecka o szczepieniach ochronnych spoczywa tylko i wyłącznie na lekarzu sprawującym profilaktyczną opiekę zdrowotną. (art.17 ust. 9 Ustawy z dnia 5 grudnia 2008 r. o zapobieganiu oraz zwalczaniu zakażeń i chorób zakaźnych u ludzi). Pozostali pracownicy ochrony zdrowia (położne, pielęgniarki, lekarze ginekolodzy i położnicy, lekarze oddziałów noworodkowych, lekarze specjaliści sprawujący opiekę nad dzieckiem w pierwszych dniach, tygodniach lub miesiącach życia) nie są w Polsce prawnie zobowiązani do informowania rodziców/opiekunów dziecka o szczepieniach ochronnych zarówno obowiązkowych, jak i zalecanych.

Zapewnienie rodzicom/opiekunom dziecka pełnej, satysfakcjonującej ich informacji na temat obowiązującej profilaktyki chorób zakaźnych i przeprowadzenie z nimi rozmowy rozwiewającej wszystkie ewentualne wątpliwości dotyczące szczepień wymaga jednak czasu. A to jest element deficytowy w wszystkich publicznych systemach opieki zdrowotnej. Według danych amerykańskich ponad połowa (53%) lekarzy poświęca zaledwie 10-19 minut na przeprowadzenie dyskusji na temat szczepień z rodzicami swych pacjentów, a tylko 8% lekarzy spędza na takich rozmowach ponad 20 minut [18].

Także lekarze zatrudnieni w obecnym systemie podstawowej opieki zdrowotnej w Polsce nie są w stanie zapewnić pacjentom odpowiedniego czasu do wyjaśnienia podstawowych problemów profilaktyki chorób zakaźnych. Podnoszony jest także problem dopuszczalności wykluczenia z grona pacjentów dziecka, którego rodzice odmawiają uporczywie realizacji Programu Szczepień Ochronnych. Decyzje takie są uzasadniane brakiem porozumienia i zaufania na linii lekarz – pacjent. Zjawisko to narasta także wśród lekarzy amerykańskich. Problem ten rodzi obawy co do ewentualnej kumulacji pacjentów sprzeciwiających się szczepieniom pod opieką wyrozumiałych dla tych decyzji lekarzy także niechętnych szczepieniom, co może stanowić zagrożenie epidemiologiczne. Z drugiej strony decyzje o wykluczeniu mogą budzić zastrzeżenia natury etycznej [18].

## Jak rodzice oceniają szczepienia?

Opinie rodziców na temat szczepień ochronnych stanowiły przedmiot wielu badań na przestrzeni ostatnich lat. Jednym z nich jest praca publikowana w *Przeglądzie Epidemiologicznym* przez zespół ekspertów Narodowego Instytutu Zdrowia Publicznego – Państwowego Zakładu Higieny w Warszawie, z której wynika, że 98% rodziców nie wyraziło sprzeciwu co do szczepienia dziecka, a ewentualne wątpliwości wiązały się jedynie z niektórymi szczepieniami obowiązkowymi i zalecanymi z uwagi na obawy co do wystąpienia niepożądanych odczynów poszczepiennych. Obawy te dotyczyły także nadmiernej liczby szczepień podawanych w krótkim czasie. Większe zastrzeżenia dotyczące bezpieczeństwa stosowanych szczepionek wyrażali rodzice starsi wiekiem, mieszkańcy miast i lepiej wykształceni. W omawianej pracy przeprowadzonej na grupie 1045 respondentów, w wypadku 18 osób (1,7%) negatywny wpływ na decyzję o zaszczepieniu dziecka miał lekarz POZ [19]. Ten sygnał powtórzył się w badaniu z ośrodka łódzkiego, które wykazało, że tylko 12,87% rodziców potwierdziło, że na temat szczepień rozmawiał z nimi lekarz POZ i odpowiedział wyczerpująco na zadane mu pytania. Natomiast 87,13% respondentów podało, że lekarz wcale nie rozmawiał z nimi na temat szczepień. Stąd nie jest zaskakujące, że badana grupa rodziców z Łodzi, jako źródło informacji na temat szczepień wskazała internet i znajomych [20].

Dwa ważne badania przeprowadziło w latach 2013 i 2017 Centrum Badania Opinii Społecznej (CBOS).

Pierwsze z badań (2013) wykazało przychylne nastawienie do szczepień ochronnych dzieci u 79% badanych, przy istniejącym przekonaniu, że dzięki szczepieniom nie występuje wiele groźnych chorób (82%) i że ta forma profilaktyki jest najskuteczniejszą z form ochronnych dzieci przed chorobami (79%). Jednocześnie 40% ankietowanych uważa, że promocja szczepień nie wynika z rzeczywistej potrzeby, lecz jest związana z interesem koncernów farmaceutycznych, a 39% badanych nie zgadza się z tym poglądem. 73% badanych uważała, że część szczepień powinna być obowiązkowa, a 23% stało na stanowisku, że powinna obowiązywać całkowita dobrowolność szczepień [21].

W badaniu z 2017 roku, jedynie 3% Polaków odmówiło co najmniej jeden raz poddania dziecka szczepieniom obowiązkowym, a najczęstszym powodem (40%) była obawa przed niepożądanymi odczynami poszczepiennymi. Niewykonanie szczepienia obowiązkowego najczęściej deklarują młode matki (24-35 lat). Tylko niespełna połowa badanych (47%) pozytywnie ocenia poziom informacji pozyskiwanych przez rodziców na temat niepożądanych skutków działania szczepionek. Negatywne lub sceptyczne nastawienie do szczepień zadeklarowały osoby o niższym wykształceniu, gorszym statusie zawodowym i materialnym. Większe poparcie dla zniesienia obowiązku szczepienia wyrażały osoby oceniające swoje warunki materialne jako złe.

Z badania wynika też, że Polacy nie oczekują likwidacji obowiązkowych szczepień ochronnych u dzieci, co więcej zdecydowana większość badanych pozytywnie ocenia skutki prowadzonej profilaktyki i chce utrzymania ich obowiązkowego statusu. Obowiązujące przepisy, dające możliwość stosowania grzywny wobec rodziców uchylających się od szczepień, nie są w pełni społecznie akceptowane, choć w ostatnim czasie takie rozwiązane wprowadzono we Włoszech zmagających się ze wzrostem liczby zachorowań na odrę. Polacy, zwłaszcza posiadający dzieci, oczekują rozwiązań skierowanych na ograniczenie kontaktów dzieci nieszczepionych z zaszczepionymi. Zebrane dane wskazują również na luki w wiedzy rodziców na temat profilaktyki chorób zakaźnych i pilną potrzebę uzupełnienia ich wiedzy na temat bezpieczeństwa szczepień [22].

## Wnioski

1. Oceniając nasz system szczepień ochronnych w aspekcie prawnym, należy zgodzić się z opinią, że obowiązujące przepisy powinny być doprecyzowane celem zwiększenia ich przejrzystości, szczególnie pod kątem zapewnienia zarówno korzyści indywidualnych, jak i zbiorowych z realizacji Programu Szczepień Ochronnych. Wymagają szczególnie dopracowania zasady odpowiedzialności indywidualnej rodziców i opiekunów dzieci za uchylanie się od obowiązkowych szczepień ochronnych. Naruszanie tych przepisów spotyka się bowiem coraz częściej z dezaprobatą większości poddającej swoje dzieci szczepieniom obowiązkowym i zalecanym [28].
2. Dane wynikające z ostatnich badań CBOS wskazują także na potrzebę lepszej informacji i edukacji rodziców lub opiekunów dzieci ze strony pracowników ochrony zdrowia, co wiąże się z koniecznością poprawy przygotowania kadr medycznych do sprostania tym oczekiwaniom.
3. Opracowaniu nowej pełniejszej strategii polityki informacyjnej na temat szczepień ochronnych służyć powinno osiem postawionych wyżej pytań (problemów), dzięki analizie których można ustalić przyczyny negatywnego stosunku do szczepień ochronnych ze strony rodziców/opiekunów dzieci. Wśród tych problemów z pewnością należy zwrócić uwagę na konieczność podejmowania bardziej zdecydowanej reakcji na krążące wokół nas, negatywne doniesienia medialne, pogłoski lub pozbawione podstaw naukowych opinie na temat szczepień.
4. Prawny obowiązek przekazywania rzetelnej wiedzy na temat szczepień nie może także obciążać wyłącznie lekarzy podstawowej opieki zdrowotnej. Do koalicji na rzecz czynnej profilaktyki chorób zakaźnych należy instytucjonalnie włączyć także położne i pielęgniarki, a ponadto zapewnić wsparcie ze strony lekarzy innych specjalności mających codzienny kontakt z niemowlętami i małymi dziećmi oraz ich rodzicami.
5. Wskazane jest poszerzenie programu kształcenia o problematykę szczepień ochronnych już na poziomie studiów medycznych wszystkich kierunków, których absolwenci mogą mieć w przyszłości wpływ na podejmowane w tym zakresie decyzje indywidualne rodziców lub opiekunów dzieci objętych programem szczepień.
